# Effect of COVID-19 Pandemic on Physical Activity Habits, Musculoskeletal Pain, and Mood of Healthcare Workers

**DOI:** 10.14744/SEMB.2021.87523

**Published:** 2021-12-29

**Authors:** Enes Efe Is, Ali Sahillioglu, Sefa Demirel, Banu Kuran, Haci Mustafa Ozdemir

**Affiliations:** 1. Department of Physical Medicine and Rehabilitation, University of Health Sciences Turkey, Sisli Hamidiye Etfal Teaching and Research Hospital, Istanbul Turkey; 2. Department of Orthopedics and Traumatology, University of Health Sciences Turkey, Sisli Hamidiye Etfal Teaching and Research Hospital, Istanbul Turkey

**Keywords:** COVID-19, healthcare worker, mood, musculoskeletal pain, physical activity

## Abstract

**Objectives:**

Self-isolation seems to be the best way to slow down the coronavirus disease 2019 (COVID-19) outbreak, but it may also have negative impact on physical and mental health. The aim of this study was to investigate the changes in physical activity habits during the outbreak and also the impact of the pandemic on musculoskeletal pain and mood in correlation with physical activity in healthcare workers.

**Methods:**

This study is conducted through Google Forms web survey platform. A total of 310 hospital staffs completed the Google Forms questionnaire in 2 weeks during lockdown and curfew period in Istanbul. The questionnaire included 60 questions including demographic, occupational, COVID-19 exposure data, physical activity habits, musculoskeletal pain, and mood.

**Results:**

There was a significant difference between physical activity habits before and after the pandemic (p<0.001). Individuals engaged in regular physical activity (regardless of duration) had significantly higher happiness ratings (p=0.002). No statistically significant difference was found between the duration of physical activity and the musculoskeletal pain during the pandemic.

**Conclusion:**

Pandemic caused a decrease in physical activity, an unhappy and anxious mood, and an increase in musculoskeletal pain of healthcare workers. Participants who were doing regular physical activity were less unhappy, but no relationship between exercise and musculoskeletal pain was found which might be related to psychosocial state of the participants who worked under great stress with high effort during the pandemic.

Since the identification of the first case in December 2019 in Wuhan (Hubei, China), the coronavirus disease 2019 (COVID-19) has spread rapidly throughout the world and resulted in an ongoing pandemic. Turkey, with the first confirmed case on March 11, 2020, is also seriously affected by the outbreak like the rest of the world.^[1]^ Many countries, among them Turkey, took prompt and radical public health measures to slow down the contagion, especially in the beginning phase including curfews, travel restrictions, and calls for self-isolation which profoundly changed our lives in many ways. More than 4 billion people around the world were called on compulsory or recommended to remain at home to fight the outbreak.^[2]^

Quarantine and self-isolation seem to be the best ways to slow down the outbreak, but it may also have negative impact on the isolated ones’ physical and mental health status. COVID-19 threatened populations with several aspects regarding immobilization and lack of physical activity. At least 150 min of moderate-intensity physical activity (with bouts lasting >10 min) in a week is recommended for adults by the World Health Organization.^[3]^ The health benefits of physical activity and exercise include prevention of multiple diseases such as diabetes, cardiovascular diseases, osteoporosis, some cancer types, obesity both primarily and secondary, as well as preserving the cognitive and psychological wellness of the individual.^[3-11]^ Data from Hong Kong flu epidemic in 1997 show that regular exercisers had a lower mortality rate.^[12]^ Moderate-intensity exercise-induced immunomodulation might also have an important role to slow the progression of the disease.^[13-15]^

The aim of this study was to investigate the changes in physical activity habits during the outbreak of COVID-19 and the emotional impact of the pandemic in correlation with physical activity in healthcare workers who were under a big pressure because they had to fight the virus in the hospital and isolation at home.

## Methods

This study is conducted through Google Forms web survey platform. The survey focused on the healthcare workers of Sisli Hamidiye Etfal Teaching and Research Hospital . Online survey was approved by the Clinical Trials Approval Office of the Ministry of Health and local hospital ethics committee (June 30, 2020/2861). It was distributed by the hospital administration and chief doctors of the departments. The participants were informed about the purpose of the research that it was approved by the ministry and the administration. They were also informed that participation was voluntary and his/her personal information would be confidential.

A total of 310 hospital staffs completed the Google Forms questionnaire in 2 weeks during lockdown and curfew period in Istanbul.

The questionnaire included 60 questions including demographic, occupational, COVID-19 exposure data, physical activity habits, musculoskeletal pain, and mood changes that were commonly experienced during disasters. Aerobic physical activity (with bouts lasting >10 min) duration before and during the pandemic was questioned as none, <150 min, or >150 min each week. To reveal the effects of staying at home and the individual’s preferences for physical activity more clearly, physical activity time while working in the hospital were not included in this duration. Change in physical activity habits in relation with the pandemic was also questioned. Musculoskeletal pain and its relation with physical activity status before and during the pandemic were assessed through a numeric scale (0: No pain, 1: Pain did not increase, 2: Pain slightly, 3: Moderately, and 4: Extremely increased). Happiness was rated according to the respondent’s perception in nine levels (1: Extremely, 2: Very, 3 Moderately, 4: Slightly unhappy, 5: Neutral, 6: Slightly, 7: Moderately, 8: Very, and 9: Extremely happy) and anxiety in five levels (1: Not anxious at all, 2: Slightly, 3: Moderately, 4: Very, and 5: Extremely anxious). In this article, individuals with a weekly physical activity duration of “0” min are called “non-exercisers” and individuals with duration of regular physical activity above this number were referred to as “regular exercisers.”

### Statistical Analysis

Statistical analysis was performed using SPSS v.21 (SPSS Inc., Chicago, IL, USA). Continuous data are presented as the mean±standard deviation and categorical data are presented as frequency and percentage. In the analysis of categorical variables, McNemar and marginal homogeneity tests were used for pre- and post-pandemic evaluations, and Chi-square and Fisher’s exact tests were used where appropriate for other comparisons. In comparison of continuous variables, Mann–Whitney U-test was used for two groups and Kruskal–Wallis test was used for more than 2 groups. P<0.05 was considered to be statistically significant.

## Results

Table 1 shows characteristics of the participants. The study sample (n=310) included 132 men (42.6%) and 178 (57.4%) women. About 61% (n=189) were <39 years old. About 41.9% (n=130) lived with his/her spouse and child(ren). Participants were 20.6% (n=63) residents, 19.6% (n=60) nurses, 17% (n=52) specialists, and 27.8% (n=36.6) were personnel (technicians, security, etc.). While 42.5% (n=132) were working in non-surgical departments, 25.5% (n=79) were working at surgical departments. About 34.2% (n=106) were working for more than 15 years while 35.2% (n=109) for <5 years.

**Table 1.** Demographic and occupational data of the participants

**Variable**	**n (%)**
Sex	
Male	132 (42.6)
Female	178 (57.4)
Age (year)	
<30	109 (35.2)
30–39	80 (25.8)
40–49	70 (22.6)
50–59	47 (15.2)
60–69	4 (1.3)
Who do you live with?	
Alone	57 (18.4)
Spouse/partner	54 (17.4)
Spouse and child(ren)	130 (41.9)
Parents	29 (9.4)
Other	40 (12.9)
Title	
Staff	58 (19)
Technician	27 (8.8)
Nurse	60 (19.6)
Physiotherapist	12 (3.9)
Resident	63 (20.6)
Specialist	52 (17)
Associate professor	21 (6.9)
Professor	13 (4.2)
Department	
Internal medicine specialty	132 (42.5)
Surgical specialty	79 (25.5)
Laboratory	31 (10.0)
Secretary	30 (9.7)
Imaging	25 (8.1)
Other	13 (4.2)
Years in profession	
<5	109 (35.2)
5–10	50 (16.1)
10–15	45 (14.5)
>15	106 (34.2)

About 35.5% (n=110) of the participants had more than 5 nightshifts during the pandemic. About 53.5% (n=166) had a history of contact with a COVID-19 (+) patient. About 70.3% (n=218) were not tested for COVID-19. While 22% (n=67) had chest computed tomography (CT) and 29.5% (n=93) had swab sample performed, only 5.2% (16) of participants were diagnosed as COVID-19 (+). About 12.9% (n=40) were quarantined for 14 days.

Musculoskeletal pain frequency was compared among those participants who used to do regular physical activity for <150 min/week and >150 min/week or who did not engage in regular physical activity. With respect to musculoskeletal pain before COVID-19 quarantine, there was no significant difference between three groups. To understand whether there is a duration-based or independent relationship between pain and physical activity, further analyses were conducted. Regular exercisers (<150 min/week + >150 min/week) and non-exercisers and people who do physical activity >150 min/week and who do physical activity <150 min/week (non-exercisers are included in this group) were compared to investigate the effect of the cutoff value “150 min.” No significant difference was found between these groups. And regardless of physical activity, 136 (66.0%) out of 206 participants with musculoskeletal pain reported increased pain.

There was a significant difference between the physical activity habits of the participants before and after the pandemic (p<0.001). While 28.1% of those who did not engage in regular physical activity before the pandemic started to do physical activity <150 min/week during the pandemic and 6.5% started to do physical activity more than 150 min/week. On the contrary, 37.8% of those who did physical activity more than 150 min a day before the pandemic quitted regular physical activity and became non-exercisers, while 38.7% started to do physical activity for <150 min/week.

Comparing the physical activity habits of the participants before and after the pandemic in two groups (>150 min/week vs. <150 min/week + non-exercisers) with the McNemar test, we concluded that the physical activity habits of the participants had changed. We determined that 75.7% of the participants who did physical activity >150 min/week previously (n=111 people) tend to do physical activity less during the pandemic. We found that 10.6% of those who did physical activity <150 min/week before the pandemic started doing physical activity for more than 150 min/week. We commented that the participants started to do physical activity less than before during the pandemic, since a larger part of the participants started to do physical activity less. The aforementioned findings are summarized in Tables 2-4.

**Table 2.** Evaluation of physical activity habits in terms of physical activity time before the pandemic

**Physical activity during home stay**	**Physical activity per week before the pandemic**			**P**
	**Non-exerciser**	**<150 min/week**	**>150 min/week**	
Non-exerciser	91 (65.5)	21 (35.0)	42 (37.8)	<0.001
<150 min/week	39 (28.1)	27 (45.0)	42 (37.8)	
>150 min/week	9 (6.5)	12 (20.0)	27 (24.3)	

Descriptive statistics were given as number (%). *Marginal homogeneity test.

**Table 3.** Evaluation of physical activity habits in terms of regular physical activity before the pandemic (time independent)

**Physical activity during home stay**	**Engaged in regular physical activity before the pandemic (time independent)**		
	**Yes**	**No**	**P**
Yes	108 (63.1)	48 (34.5)	0.024
No	63 (36.8)	91 (65.5)	

Descriptive statistics were given as number (%). *McNemar test.

**Table 4.** Evaluation of physical activity in terms of physical activity time before the pandemic

**Physical activity during home stay**	**Physical activity time per week before the pandemic**		**P**
	**<150 min/week**	**>150 min week**	
<150 min/week	178 (89.4)	84 (75.7)	<0.001
>150 min/week	21 (10.6)	27 (24.3)	

Descriptive statistics were given as number (%). *McNemar test.

No statistically significant difference was found in terms of relationship between the duration of physical activity during the pandemic (during the home stay) and the musculoskeletal system pain during the home stay among groups.

Participants’ physical activity status and its relationship with their happiness and anxiety parameters were also evaluated and compared. In this evaluation, both physical activity – happiness, physical activity – anxiety relationships (correlation), and the difference between groups (non-exercisers vs. <150 min/week + >150 min/week and non-exercisers vs. <150 min/week vs. >150 min/week) were analyzed. In the correlation analysis, only a weak correlation was found between post-pandemic physical activity duration (no exercise vs. <150 min/week vs. > 150 min) and happiness (coefficient = 0.205). In other words, as the duration of physical activity increased, the happiness scores also increased.

When the happiness ratings between the participants who did physical activity (<150 min/week + >150 min/week) and did not engage in regular physical activity after the pandemic were compared, the happiness scores of those who did physical activity were significantly higher (p=0.002). When the happiness ratings of the participants were examined according to the duration of physical activity, a statistically significant difference was found between the groups in post-pandemic setting (p=0.007). Happiness score was found lower in non-exercisers group. These findings are summarized in Table 5.

**Table 5.** Evaluation of mood in terms of physical activity habits

	**Happiness**		**Anxiety**	
	**Mean±SD**	**P**	**Mean±SD**	**P**
Regular physical activity before pandemic		0.065^a^		0.377^a^
No (n=139)	3.6±2.4		3.4±1.3	
Yes (n=171)	4.2±2.4		3.6±1.2	
Regular physical activity during home stay		0.002^a^		0.367^a^
No (n=154)	3.5±2.3		3.6±1.3	
Yes (n=156)	4.3±2.4		3.4±1.3	
Regular physical activity before pandemic		0.077^b^		0.146^b^
No (n=139)	3.6±2.4		3.4±1.3	
<150 min/week (n=60)	4.5±2.4		3.3±1.3	
>150 min/week (n=111)	4±2.4		3.7±1.2	
Regular physical activity during home stay		0.007^b*^		0.651^b^
No (n=154)	3.5±2.3		3.6±1.3	
<150 min/week (n=108)	4.2±2.4		3.4±1.3	
>150 min/week (n=48)	4.6±2.5		3.5±1.3	

^a^Mann–Whitney U-test, ^b^Kruskal–Wallis test. *Non-exercisers group is different from other groups. SD: Standard deviation.

## Discussion

The results of our study demonstrated that pandemic has changed people’s, in our study healthcare worker, physical activity habits. Some of the people who do physical activity for more than 150 min/week tend to do less or quitted regular physical activity completely. Some of the people who did not engage in regular physical activity before the pandemic started to do physical activity during lockdown. However, physical activity time has decreased due to pandemic, in other words due to restrictions as expected. Walking time >10 min is also included in our study as aerobic exercise/physical activity. Therefore, it can be concluded that our results are consistent with a previous descriptive study using smartphones to track daily step counts. Tison et al.^[16]^ reported a rapid worldwide decrease up to 48.7% in step counts, especially in regions with lockdowns. Górnicka et al.^[17]^ also reported a reduction over 40% in physical activity as a result of their similar designed electronic survey with 2381 respondents during quarantine in Poland. In our study, 33.9% (105/310) of the participants reported reduced physical activity. The reduction of exercise time might be explained through the inability to turn home to a gym for various reasons^[18]^ and through anxiety levels, but in healthcare workers’ case, increased physical and emotional stress cannot be ignored.

Although we accept 150 min/week as the threshold value for the purpose of separating physically active and inactive individuals in further analyzes in our study, this value is also controversial in the literature.^[19]^ Although this threshold value is based on studies related to the prevention of various diseases and mortality,^[20,21]^ there is no optimal agreed aerobic exercise/physical activity time threshold value in the literature that has been shown to have an effect on musculoskeletal pain intensity and happiness and anxiety levels that we investigated in our study. However, given that painful individuals may have less exercise tolerance, the recommended 150 min/week threshold for general health benefits may be high. Aerobic physical activity intensity was also not questioned in detail, but we mainly focused on physical activity time which could be a limitation of our study.

Exercise has a very important role in musculoskeletal pain. Evidence showing the positive effects of regular exercise on pain is increasing, especially in patients with chronic pain.^[22]^ In a meta-analysis investigating the effectiveness of walking-based interventions in chronic musculoskeletal pain, it was demonstrated that aerobic exercise could improve pain.^[23]^ It is a limitation of our study that pain duration is not questioned in detail. However, similar to ours, in a Korean study that grouped the participants as active and inactive individuals over the 150 min/week threshold, and, unlike our study, investigated the elderly female population, it was stated that the inactive group generally experienced more serious problems related to pain.^[24]^ In our study, there was no difference in musculoskeletal pain between individuals who do physical activity regularly and those who do not, even in further analysis with 150 min/week threshold.

However, in studies investigating the relationship between psychosocial status and pain, it has been reported that psychosocial problems have a negative effect on parameters related to pain.^[25-31]^ Similarly, it is known that social isolation, as a natural consequence of restrictions, has a negative effect on pain and physical activity level too.^[32-35]^ In a study conducted in our country at the same time as our study, in which healthcare workers, mostly nurses, were evaluated, it was found that approximately one-third of the participants experienced severe extremely severe depression and anxiety, and a quarter of them were under severe extremely severe stress.^[36]^ In a study conducted in Spain, in which individuals with chronic pain (52.2% musculoskeletal pain) were included, most of the participants stated that their pain increased,^[37]^ as in our study. Although no significant relationship with physical activity status was found in our study, people tend to suffer more from the musculoskeletal pain. Evaluating the data together, the psychosocial state and workload of health-care professionals during the pandemic can be associated with the increase in pain. Furthermore, failure to determine a relationship between physical activity status and pain may be due to these psychosocial factors. Another limitation of our study is that we used a numeric scale instead of common generic scales for the evaluation of depression and anxiety, and this may have reduced inference efficiency.

While there was no relationship between physical activity status before the pandemic and happiness, doing physical activity during home stay was associated with higher happiness ratings and did not depend on physical activity duration. However, there is no relationship between anxiety and physical activity before and after the pandemic. In general, considering the mean values, it was observed that the participants were mostly unhappy and very anxious as in the study of Alan et al.^[36]^ In a survey study investigating the effects of the pandemic on the mental health of medical students and newly qualified doctors in the UK, the mood of those who exercise to maintain their mental well-being was better similar to our study.^[38]^ As a result of a large-scale study investigating the exercise parameters to prevent depression, it was stated that the protective effect occurs at low level of physical activity (60 min/week) and is independent of intensity.^[39]^ This result is consistent with our findings that the participants in our study who do physical activity regularly, regardless of the physical activity duration threshold 150 min/week, were less unhappy.

The main limitations of the present study are the absence of the details related to physical activity intensity and pain duration. The non-use of generic scales for depression and anxiety and also for physical activity, as well as the exercise duration threshold value “150 min/week,” might affect the results. Another limitation of our study is that the physical activity performed in the hospital was not included in the total weekly duration, and therefore, for some participants, the survey may have shown the weekly physical activity time shorter than it actually was.

## Conclusion

Pandemic caused a decrease in physical activity, an unhappy and anxious mood, and an increase in musculoskeletal pain of healthcare workers. Regular exercisers were less unhappy, but no relationship between physical activity and musculoskeletal pain was found which might be related to psychosocial state of the participants.

### Disclosures

**Ethics Committee Approval:** The study was approved by the Local Ethics Committee of University of Health Sciences, Sisli Hamidiye Etfal Teaching and Research Hospital (June 30, 2020/2861).

**Peer-review:** Externally peer-reviewed.

**Conflict of Interest:** None declared.

**Authorship Contributions:** Concept – E.E.I., B.K., H.M.O.; Design – E.E.I., B.K.; Supervision – B.K.; Materials – E.E.I., S.D.; Data collection &/or processing – E.E.I, S.D.; Analysis and/or interpretation – E.E.I., A.S.; Literature search – E.E.I, A.S., S.D.; Writing – E.E.I., A.S.; Critical review – B.K., H.M.O.
